# Feasibility and Acceptability of a Wearable Technology Physical Activity Intervention With Telephone Counseling for Mid-Aged and Older Adults: A Randomized Controlled Pilot Trial

**DOI:** 10.2196/mhealth.6967

**Published:** 2017-03-06

**Authors:** Elizabeth J Lyons, Maria C Swartz, Zakkoyya H Lewis, Eloisa Martinez, Kristofer Jennings

**Affiliations:** ^1^ Department of Nutrition and Metabolism The University of Texas Medical Branch Galveston, TX United States; ^2^ Sealy Center on Aging The University of Texas Medical Branch Galveston, TX United States; ^3^ Division of Rehabilitation Sciences The University of Texas Medical Branch Galveston, TX United States; ^4^ BeachBody, LLC Santa Monica, CA United States; ^5^ Department of Preventive Medicine and Community Health The University of Texas Medical Branch Galveston, TX United States

**Keywords:** physical activity, technology, mobile health, health behavior, self-control

## Abstract

**Background:**

As adults age, their physical activity decreases and sedentary behavior increases, leading to increased risk of negative health outcomes. Wearable electronic activity monitors have shown promise for delivering effective behavior change techniques. However, little is known about the feasibility and acceptability of non-Fitbit wearables (Fitbit, Inc, San Francisco, California) combined with telephone counseling among adults aged more than 55 years.

**Objective:**

The purpose of our study was to determine the feasibility, acceptability, and effect on physical activity of an intervention combining a wearable physical activity monitor, tablet device, and telephone counseling among adults aged 55-79 years.

**Methods:**

Adults (N=40, aged 55-79 years, body mass index=25-35, <60 min of activity per week) were randomized to receive a 12-week intervention or to a wait list control. Intervention participants received a Jawbone Up24 monitor, a tablet with the Jawbone Up app installed, and brief weekly telephone counseling. Participants set daily and weekly step goals and used the monitor’s idle alert to notify them when they were sedentary for more than 1 h. Interventionists provided brief counseling once per week by telephone. Feasibility was measured using observation and study records, and acceptability was measured by self-report using validated items. Physical activity and sedentary time were measured using ActivPAL monitors following standard protocols. Body composition was measured using dual-energy x-ray absorptiometry scans, and fitness was measured using a 6-min walk test.

**Results:**

Participants were 61.48 years old (SD 5.60), 85% (34/40) female, 65% (26/40) white. Average activity monitor wear time was 81.85 (SD 3.73) of 90 days. Of the 20 Up24 monitors, 5 were reported broken and 1 lost. No related adverse events were reported. Acceptability items were rated at least 4 on a scale of 1-5. Effect sizes for most outcomes were small, including stepping time per day (d=0.35), steps per day (d=0.26), sitting time per day (d=0.21), body fat (d=0.17), and weight (d=0.33).

**Conclusions:**

The intervention was feasible and acceptable in this population. Effect sizes were similar to the sizes found using other wearable electronic activity monitors, indicating that when combined with telephone counseling, wearable activity monitors are a potentially effective tool for increasing physical activity and decreasing sedentary behavior.

**Trial registration:**

Clinicaltrials.gov NCT01869348; https://clinicaltrials.gov/ct2/show/NCT01869348 (Archived by WebCite at http://www.webcitation.org/6odlIolqy)

## Introduction

### Background

Increasing physical activity and decreasing sedentary behavior can reduce the risk of many negative health outcomes among older adults, including cardiovascular diseases, Type II diabetes, cancer, and all-cause mortality [1-4]. The effects of moderate-vigorous intensity physical activity and sedentary behavior on these outcomes appear to be independent [5,6]; thus, older adults could benefit from interventions targeting both behaviors simultaneously. Unfortunately, rates of physical activity are low in this population. Recent estimates from objective monitoring suggest that most American adults spend less than 2% of their time in moderate-vigorous intensity physical activity [7]. Moderate-vigorous intensity activity decreases with age [8]; improving activity habits among mid-aged and older adults could prevent later functional decline and even mortality [9,10]. In addition, sedentary behavior is highly prevalent, amounting to most of adults’ waking hours [11]. Although interventions have demonstrated positive effects on both behaviors, these methods suffer from limitations related to poor sustainability and poor scalability [12]. There is a clear need for interventions that are effective in the long term as well as the ones that are easy to disseminate.

Wearable electronic activity monitors are advanced versions of pedometers that are able to offer more behavior change techniques and implement them in different ways as compared with standard displays on the device itself [13]. Because these devices send information to a mobile app, they are able to offer feedback that better conforms to theoretical recommendations (eg, specific, clear, and comparing with similar others; past accomplishments; and specific goals) [14,15]. They also deliver many additional behavior change techniques that are not possible with standard pedometers, such as goal setting, social support, and cues to action. Cues to action are likely particularly important for replacing sedentary behavior with physical activity, as they alert participants to their sedentary behavior in real time. Traditionally, delivery of these behavior change techniques would require either in-person counseling or frequent (and thus expensive) tailored print materials. Delivery via mobile app offers an opportunity for interventions both effective and with broad reach.

These improvements on pedometers show promise, but wearable electronic monitors and their companion mobile apps still lack several important behavior change techniques. In particular, empirically proven techniques such as action planning and problem solving are typically absent from these apps [15]. Adding brief counseling to provision of these devices could allow interventionists to deliver the full range of behavior change techniques standard in behavior physical activity interventions. The counseling should provide any techniques missing from the apps, while the apps allow for improved implementation of other fundamental techniques.

Studies of wearable electronic devices and mobile apps published thus far have found equivocal physical activity outcomes, though their use of Fitbit and Bodymedia products may not generalize to other self-monitoring systems [13,16,17]. It is also possible that these devices may not be feasible or acceptable to older adult populations [18], who are in unique need of more effective activity interventions. Feasibility and acceptability of mobile phone–based intervention among adults aged more than 55 years is not yet clear. Some studies have found low acceptability and preference for other media [18] as well as increased barriers to mobile phone use with increasing age [19]. Older adults have also reported the feeling that iPads were designed for younger audiences than for their age group [20]. However, some studies have found positive feasibility and acceptability results for smart device health interventions in this age group [21,22]. Short-term tests indicate that activity monitors may be acceptable to mid-aged and older adults [23], but the feasibility and acceptability of longer-term usage is unclear. Of the few published studies of wearable electronic activity monitors, even fewer have described in detail feasibility and acceptability results [24].

### Objective

The purpose of this study was to investigate the feasibility and acceptability of an intervention including the Jawbone Up system and telephone counseling. To our knowledge, this system is yet to be tested in intervention trials. In a content analysis of available wearables and their apps, the Up system included the most behavior change techniques and implemented them very closely to theoretical recommendations [15]. In addition to providing a wearable device and mobile app, we also provided brief weekly telephone counseling that was adapted to include behavior change techniques known to be important in physical activity research that were absent from the Up system [15,25-27]. We hypothesized that the intervention would be feasible and acceptable for this population. To operationalize these outcomes, we specifically measured days the monitor was worn and self-reported acceptability items taken from similar eHealth studies.

## Methods

### Recruitment

**Figure 1:**
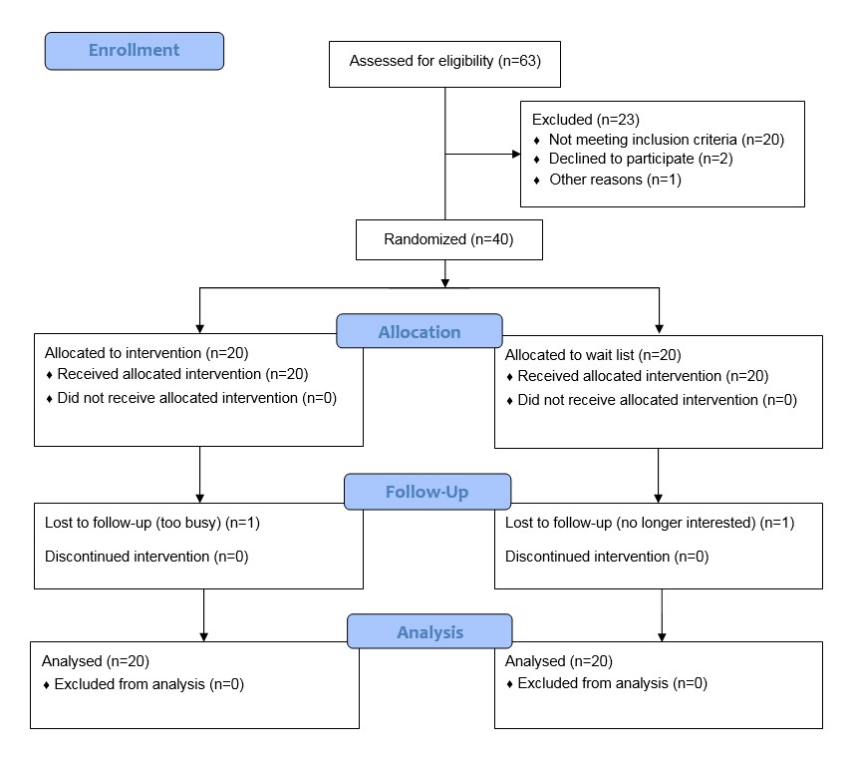
CONSORT diagram.

Figure 1 shows the CONSORT diagram for the study. This trial was a parallel randomized controlled pilot trial with 1:1 group allocation. Participants (N=40) were recruited in 2 cohorts of 20 between 2014 and 2015 via advertisements in local newspapers, online mailing lists, and university announcements. Cohort 1 was recruited over 6 months in 2014, and cohort 2 was recruited over 8 months in 2015. Major inclusion criteria were ages between 55 and 79 years, body mass index (BMI) between 25 and 35, the ability to read and understand English, and the ability to read words on a tablet-sized device. Major exclusion criteria included self-reported habitual physical activity more than 60 min per week, health issues that might preclude safe walking, psychological issues that might interfere with full participation, current use of a wearable electronic activity monitoring system, and endorsing cardiovascular risk questions on the Physical Activity Readiness Questionnaire [28]. If the only questions endorsed had to do with taking medication, individuals could participate if they provided a doctor’s consent. Randomization was conducted using standard opaque envelopes with foil (to prevent seeing the group assignment inside the envelope) and carbon paper (to provide an audit trail). The envelopes were randomly sorted by an individual not involved with the randomization visit process, then numbered sequentially. As interventionists opened each envelope, they signed and dated each envelope and saved the inner paper with original printed allocation and carbon-copied sequence number, ID number of the participant, signature of interventionist, and date of opening. Randomization was carried out using sequentially numbered opaque sealed envelopes according to standard protocols [29], with randomization stratified by the 2 cohorts to promote adequate numbers of participants able to talk to one another through the app.

### Procedure

Participants attended 4 scheduled visits. The first consisted of informed consent procedures and provision of a research-grade activity monitor. Participants were provided information on the intervention procedures and nature of the wearable and app prior to providing informed consent. Approximately a week later, participants returned for a full baseline assessment and orientation to the study. A midpoint assessment occurred at 6 weeks (questionnaire and physical activity assessment only), and a full final assessment occurred at 12 weeks. Participants could not be blinded to their group. Unfortunately, resource limitations precluded using blinded assessors for all participants. All procedures were approved by the University of Texas Medical Branch Institutional Review Board and registered at clinicaltrials.gov prior to beginning data collection. Figure 1 shows the flow of participants through the trial using a CONSORT diagram.

### Intervention

**Figure 2:**
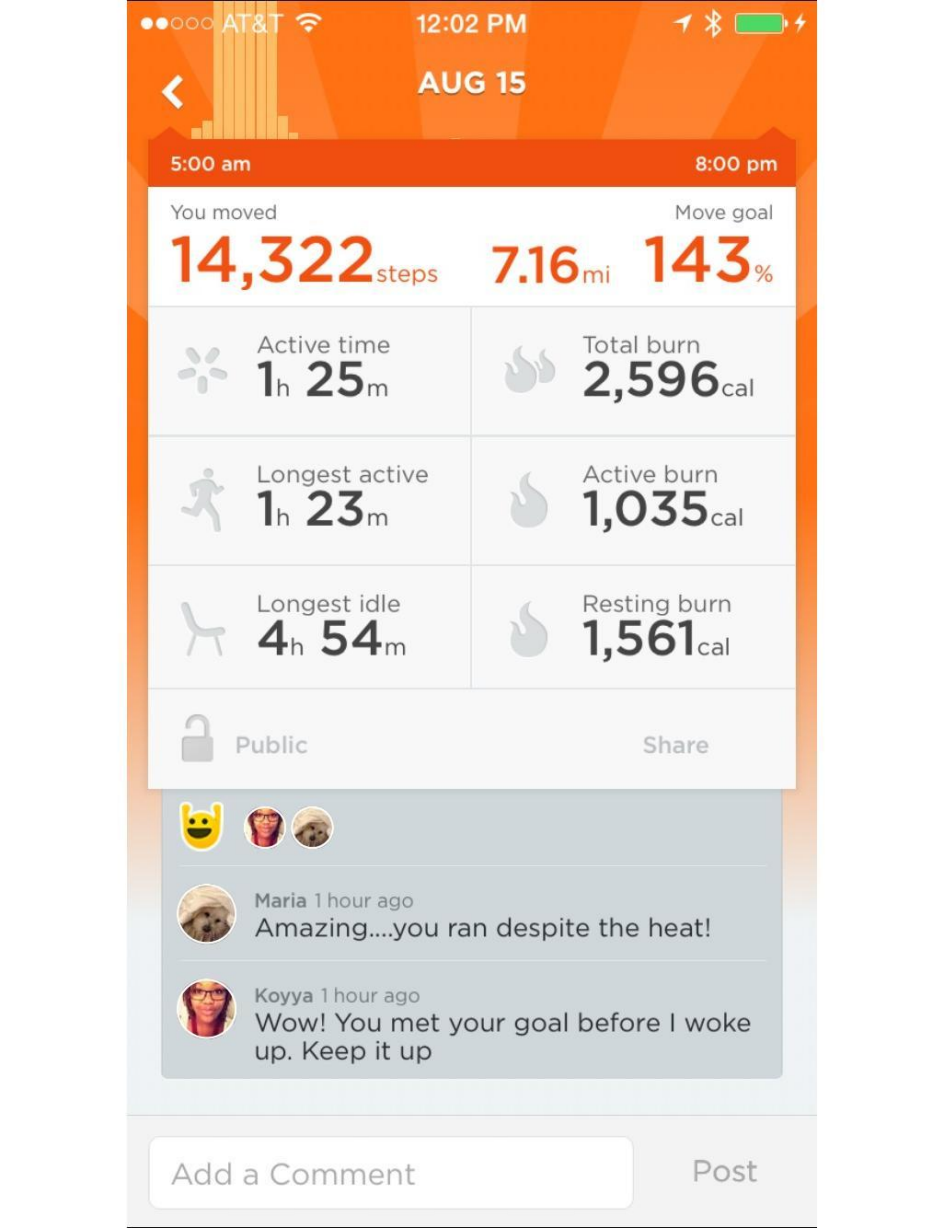
Screenshot of the Jawbone Up app.

The participants randomized to the intervention group were lent a mini tablet mobile device (Apple iPad Mini, Apple Inc, Cupertino, CA) and a wearable electronic activity monitor (Up24, Jawbone Inc, San Francisco, CA) for home use during the study. The tablet was preloaded with the Jawbone Up app and synced to an Up24 for each participant. Figure 2 shows an example of activity feedback and social support in the Up app. Please note that this example used data and social interaction from researchers in the study, not from the study participants. Detailed information on the contents of the app, including behavior change techniques and adherence to theory-based recommendations, are available in a previous publication [15]. All the participants were provided with premade accounts that existed on a “team” with all other participants as well as an account for interventionist surveillance. The orientation visit included guidance on the use of the wearable and app, encouragement to comment and like others’ activity, and an initial goal-setting session. Interventionists encouraged participants to view their data at least twice per day, in the morning and late afternoon or evening. Participants set goals for physical activity (short- and long-term) and sedentary behavior (longest bout length). Interventionists provided training for self-monitoring, viewing feedback, and using sedentary behavior prompts in the app. Although some changes in the appearance of the app and tools provided in the app occurred during the overall study period, no substantive changes to the physical activity feedback content occurred. We were unable to determine whether individual participants updated their apps during their intervention periods, but updates should not have affected the overall experience.

Weekly telephone counseling was provided by a team led by the principal investigator and a postdoctoral fellow with extensive training in behavioral counseling. The team included a predoctoral fellow and a clinical research coordinator who were trained by the principal investigator and the postdoctoral fellow. Initial calls by team members were observed by the principal investigator and postdoctoral fellow and feedback was provided to maintain quality. In addition, team members followed a scripted counseling guide. Counseling calls were designed to last approximately 15-20 min each. Each counseling call included a check-in for any adverse events or technical problems, reevaluation of weekly step goals, and action planning for the next week. Goals were negotiated between the counselors and individuals, with counselors suggesting at least 7000 steps per day (based on step counts found to be appropriate for very deconditioned older adults, as we determined with baseline fitness tests) [30] on 2 days per week, increasing over time to at least five days per week. Sedentary bout goals were also negotiated, with 1 h being the number suggested and typically agreed to by participants. These goals were entered into the app so that progress bars would measure progress toward the specific goals. Idle alerts used the sedentary bout goals to determine when to vibrate to alert participants that they had been sedentary too long.

Weekly special topics delivered additional behavior change techniques from the CALO-RE framework [31] that are as follows: planning social support, problem-solving, self-rewards, when and where to perform the behavior, relapse prevention, stress management, and time management. The app provided other behavior change techniques, listed in the Multimedia Appendix 1 (see Lyons et al [15] for more complete descriptions; only behavior change techniques related to physical activity are listed here). Because the SmartCoach portion of the app adjusted the content based on the user behavior, we cannot state with certainty which behavior change techniques were delivered to each participant by the app. Some participants may not have triggered delivery of every possible behavior change technique. Interventionists based their counseling on the data taken from the app over the last week to negotiate changing goals.

The wait-list control group did not receive any intervention until after their final assessment, when they were provided the intervention in full.

### Measures

Physical activity was measured using an ActivPAL device (PAL Technologies Ltd, Glasgow, Scotland). This small, thin device was attached to the front of each participant’s thigh, midway between the knee and the trunk, using an adhesive strip. The ActivPAL is well-validated for use in measuring physical activity as well as sedentary behavior [32,33]. Participants wore the devices for a period of 7 days at each assessment period (baseline, 6 weeks, 12 weeks). No specific criteria have been published for determining wear time and usable data for these monitors. Following the procedures of Bickmore et al [34], we removed daily activity values less than the 5th percentile (0.00 stepping minutes), as we considered it possibly representative of non-wear time (eg, time spent being mailed back and forth to participants, time being carried rather than worn). Physical activity was operationalized as mean minutes of physical activity per day, mean minutes spent sitting per day, and mean steps per day across all valid days per assessment.

Feasibility was measured in several ways. Use of the monitor was measured first by abstracting information weekly from the app. Because the interventionist account was a friend of each participant account, their daily data were posted to our news feed. We confirmed these data at the conclusion of the study using downloadable comma separated value files from the Jawbone website. These files provided extensive information as to different parts of the app that were used each day. Days in which activity, food, and sleep were logged were taken from these files. Attrition, adverse events, completed counseling calls, and reports of technical problems or loss of equipment were taken from the study records kept by interventionists (phone counseling logs) and the clinical research coordinator (records from emails and phone calls from participants). Acceptability of the monitor, app, and tablet were measured using items adapted from Vandelanotte et al [35,36]. Several additional items specific to the monitor and apps were included (eg, “I would continue using the idle alert”). These items used the same stems and responses as the ones adapted from previous research. All responses were made on a Likert-type scale from 1 (strongly disagree) to 5 (strongly agree). We also used a measure of perceived competence from the Intrinsic Motivation Inventory, with its items adapted to discuss competence using the tablet [37].

Fitness was estimated using a 6-min walk test [38]. Participants were asked to walk for 6 min in a rectangular route marked with cones, with a trained assessor tracking the time and laps completed. Once the activity was performed, participants waited where they stopped, while the assessor measured their distance from the closest cone. Distance walked in 6 min was measured in terms of feet.

Percent body fat was estimated using Dual x-ray Absorptiometry (GE Lunar iDXA, GE Medical Systems Lunar, Madison, WI) at baseline and 12 weeks. Height and weight were measured using a stadiometer and calibrated scale. Sociodemographics were recorded at the baseline and included age, gender, race, and ethnicity.

Weekly telephone counseling logs were completed by counselors to indicate whether counseling calls were completed or missed. Counselors attempted to contact participants a maximum of 5 times, if a counseling call was missed at the scheduled time.

All self-report measures were taken in-person using paper questionnaires. Several indicators of feasibility were measured using data from the mobile app, but all other assessments occurred face-to-face.

### Data Analysis

Data were analyzed using the R system version 3.3.1 (R Foundation for Statistical Computing, Vienna, Austria) [39]. Differences at baseline were investigated using Student’s *t* tests and chi-square tests. Differences between groups were estimated using analyses of covariance (ANCOVA), controlling for baseline values of the dependent variable. Box-Cox transformations were used to improve the validity of the inference. All the analyses used the intent-to-treat principle, bringing the last observation forward for the ones who dropped out. Multiple imputations were not used due to findings that data were not missing at random. An analysis of only study completers was also conducted for comparison purposes. In an attempt to account for potential clustering of effects due to social networking, we also ran models that included a random effect of cohort. Effect sizes presented are in the form of Cohen d.

As this was a pilot test intended to investigate feasibility, this study was not powered to detect a statistically significant difference in the primary outcome. Rather, the purpose of statistical tests was to provide estimated effect sizes that could inform decision making regarding development of a follow-up, fully powered intervention trial.

## Results

**Figure 3:**
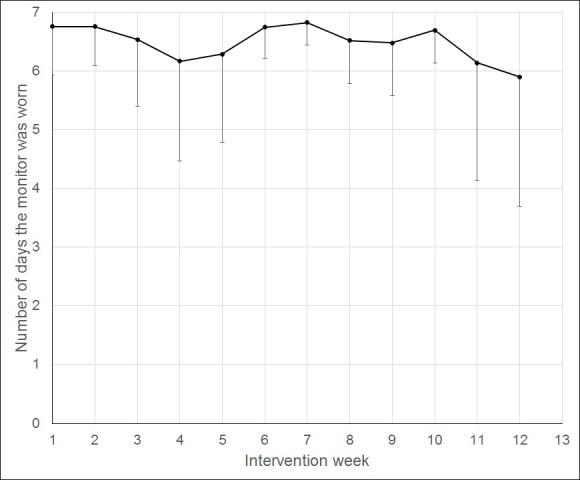
Changes in wear of the Up24 monitor by week (mean, SD).

As shown in Table 1, Participants (N=40) were 61.5 (SD 5.6) years old with a BMI of 30.3 (SD 3.5). They were mostly female (34/40, 85%) and white (26/40, 65%). No related adverse events were reported. In the intervention group, 1 participant dropped out as compared with 1 in the wait list control. Participants in the intervention group completed a mean of 10.2 (SD 2.4) of 12 counseling calls. Participants wore their Up24 monitors on average 81.85 (SD 3.73) of 90 days, with a minimum of 69 days. Although the intervention did not instruct usage of nonactivity portions of the app, participants also spontaneously tracked their sleep (Mean 11.70, SD 11.97 days) and food intake (Mean 2.65, SD 7.83 days). Figure 3 shows changes to wear of the monitor from week to week as mean and standard deviation (lower bar).

**Table 1:** Participant characteristics.

Characteristics	Intervention (n=20)	Wait list (n=20)	Total (N=40)
Age, Mean (SD)	61.25 (5.00)	61.70 (6.26)	61.48 (5.60)
Weight, Mean (SD)	82.58 (11.96)	82.14 (9.82)	82.36 (10.81)
BMI^a^, Mean (SD)	30.00 (2.86)	30.68 (4.01)	30.34 (3.45)
Female, n (%)	17 (85)	17 (85)	34 (85)
White, n (%)	13 (65)	13 (65)	26 (65)
Black, n (%)	3 (15)	2 (10)	5 (13)
Other race, n (%)	2 (10)	4 (20)	6 (15)
Hispanic ethnicity, n (%)	6 (30)	5 (25)	11 (28)
College degree, n (%)	13 (65)	14 (70)	27 (68)

^a^BMI: body mass index.

During the study period, 5 Jawbone Up24 monitors were reported broken by participants and were replaced. One additional monitor was lost and replaced. No tablets were lost, and all technical problems with them were resolved without the need for replacement. Responses to acceptability questions are shown in Table 2 **,** broken down to show responses by participants under the age of 60 years as compared with the participants 60 years or older. All but one of the questions (including reverse-coding the negatively worded question) received a mean rating over 4 of 5 across both age groups, with only one receiving a 3.9 for the participants ages 60 or above (“would you continue to wear the monitor?”). Usage and step data were successfully retrieved weekly from the Up app by research assistants. Because the interventionist account was a “friend” of each participant, participants’ information and discussions were displayed on the interventionist account news feed.

**Table 2:** Acceptability of monitor, tablet, and app.

Item	Mean scores of the participants <60, n=9, mean (SD)	Mean scores of the participants >60, n=10, mean (SD)	Mean scores of all the participants, n=19, mean (SD)
Comfort using the monitor	4.78 (0.67)	4.60 (0.52)	4.68 (0.58)
Would continue to wear the monitor	4.56 (1.33)	3.90 (1.29)	4.21 (1.32)
Comfort using the tablet	5.00 (0.00)	4.30 (1.34)	4.63 (1.01)
Tablet was user-friendly	5.00 (0.00)	4.70 (0.68)	4.84 (0.50)
Felt confident using tablet	4.44 (0.73)	4.00 (1.70)	4.21 (1.32)
Convenient to use app	4.89 (0.33)	4.60 (0.70)	4.74 (0.56)
Would like to continue to use the app	4.67(1.00)	4.40 (0.70)	4.53 (0.84)
App was user-friendly	4.89 (0.33)	4.50 (0.71)	4.68 (0.58)
Would rather use a pedometer	1.00 (0.00)	1.10 (0.32)	1.05 (0.23)
Idle alert was useful	4.56 (0.73)	4.10 (1.45)	4.32 (1.16)
Would continue using idle alert	4.33 (1.12)	4.60 (0.84)	4.47 (0.96)
Step goal was useful	5.00 (0.00)	4.90 (0.32)	4.95 (0.23)
Would continue using step goal	4.89 (0.33)	4.80 (0.63)	4.84 (0.50)
Information was credible	4.78 (0.44)	4.50 (1.08)	4.63 (0.83)
Information was relevant	4.67 (0.71)	4.60 (0.52)	4.63 (0.60)
Tips and advice were specific to me	4.33 (1.00)	4.40 (1.08)	4.37 (1.01)
Perceived competence using tablet	6.80 (0.31)	6.07 (1.53)	6.41 (1.16)

Physical activity, body composition, and anthropometric results for both groups from ANCOVA models are shown in Table 3. Analyses conducted with only the study completers with complete data did not produce substantially different results (eg, effect sizes based on only complete data were 0.40 for minutes and 0.31 for steps as compared with 0.35 and 0.26, respectively, in the intent-to-treat analysis). Therefore, we have presented the results from the intent-to-treat analysis. When we added a random effect for cohort to these models to account for potential effects of social interaction within cohorts, results did not change meaningfully. Intraclass correlation coefficients ranged from 0 (minutes sedentary, body fat) to 0.10 (weight).

**Table 3:** Physiological effects of the intervention at 12 weeks.

Outcome	Intervention		Wait list		Effect size d (95% CI)
	Baseline	12 weeks	Baseline	12 weeks	
Stepping time per day (min), mean (SD)	66.33 (23.78)	117.69 (121.37)	60.27 (25.55)	58.08 (33.03)	0.35 (0.02 to 0.68)
Steps per day, mean (SD)	5103.29 (1929.64)	6193.75 (3183.50)	4627.63 (1930.76)	4586.79 (2476.06)	0.26 (−0.07 to 0.59)
Sitting time per day, mean (SD)	1132.04 (127.19)	1088.92 (175.56)	1142.29 (129.93)	1149.44 (147.69)	−0.21 (−0.54 to 0.12)
Body fat, mean (SD)	44.98 (5.28)	44.73 (5.73)	45.17 (5.39)	45.38 (6.06)	−0.17 (−0.50 to 0.17)
Weight (kg), mean (SD)	82.58 (11.96)	81.72 (11.71)	82.14 (9.82)	82.85 (9.77)	−0.33 (−0.67 to 0.00)
Fitness (feet), mean (SD)	1742.92 (217.61)	1729.49 (296.54)	1627.39 (265.20)	1661.16 (267.57)	−0.05 (−0.39 to 0.29)

## Discussion

### Principal Findings

This physical activity and sedentary behavior intervention using an electronic activity monitor system with phone counseling was found to be feasible and acceptable in a sample of older adults. The study was also found to produce significant but small changes in total physical activity time and weight favoring the intervention group. Statistical differences between groups were not interpretable due to the underpowered nature of this trial. Effect sizes (0.35 for minutes, 0.26 for steps, 0.21 for sedentary time) suggest that a larger-scale implementation of the intervention will likely produce small but potentially clinically significant improvements in physical activity time and steps taken.

The findings of this study are very similar to the findings of a pilot study of postmenopausal women using a Fitbit system, who increased their steps from approximately 5900 at baseline to 6700 at 16 weeks [16]. Comparing Fitbit One and a brief counseling with a pedometer group, Cadmus-Bertran et al found an effect size of approximately 0.24 for steps, whereas our comparison with a wait-list control produced an effect size about approximately 0.26 (from approximately 5100 to 6200 steps, as compared with approximately 4600 steps at both time points in the control group). Another study that compared Fitbit One with texting with Fitbit One without texting found no significant difference between groups and no increase in steps compared with baseline in either group [40]. A preexperimental study of Fitbit provision among adults more than 60 years of age also found a very similar increase of approximately 1100 steps over 12 weeks [41]. A large-scale study of weight loss that compared a standard behavioral weight loss intervention with and without the use of a BodyMedia wearable monitor found no difference between the 2 on physical activity [17]. The BodyMedia monitor was substantially different from Fitbit and Jawbone in terms of behavior change techniques available in the app [15], which may partially explain this different result. Taken together, these results suggest that wearable electronic activity monitors with sophisticated feedback apps and supplemental guidance can indeed produce a clinically significant increase in physical activity.

Nearly half of our sample (18/40, 45%) self-reported as black, Hispanic, or other race, groups that are at increased risk of inactivity and related negative health outcomes. More than half of the sample (22/40, 55%) were aged above 60 years, with 10 (25%) above 65 years. Baseline fitness estimates also indicated that many of the participants were quite deconditioned. In all, this sample represents a population in critical need of novel and effective interventions to increase physical activity. Ample epidemiological evidence suggests that even small increases in physical activity among sedentary older adults can produce large health improvements [3,42]. Although a standard physical activity intervention might be expected to produce an increase of 1000-2000 steps [12], these interventions are typically much more intensive than the one tested here and thus likely more difficult to disseminate.

Feasibility and acceptability findings showed that participants overall were compliant and reported enjoying the intervention. Based on our findings for broken and lost monitors, researchers may need to purchase extra monitors for any long-term planned implementation. This study required purchase of 6 additional monitors on top of the original 20, which was more than expected. Acceptability findings were high for all components of the intervention, including for participants aged above 60 years. Despite technical issues such as broken monitors (not syncing, not powering on, buttons falling off), participants reported that the monitor, tablet, and app were user-friendly. No participant stated that they would rather use a simple pedometer instead of the provided wearable electronic activity monitor.

These results also raise many questions for future research. The extent to which apps with wearable devices cause increases in physical activity, as compared with telephone counseling or the two in combination, is not clear. We specifically designed brief counseling to address behavior change techniques absent from the app; a study of the Jawbone monitor in isolation may find different results. In addition, we arranged for participant accounts to be “friend”ed with other participants in their cohort to allow for anonymous “likes” and comments. Social interactions, either with participants or with family or friends, could be a powerful tool for increasing the efficacy of these devices.

### Limitations

The rigor of this study was limited by several aspects of its study design and by its nature as a small project conducted with very limited financial resources. As a pilot study, it was not fully powered to detect statistically significant differences in its outcomes or long-term behavior maintenance. We also cannot determine feasibility, acceptability, or effects of individual portions of the intervention such as the monitor only or telephone counseling only. Comparing with a wait-list control also limits our ability to interpret feasibility or acceptability as compared with other interventions such as pedometers. Although the effect sizes may be useful for assistance in powering future studies that use wearable electronic activity monitors, we do not report *P* values. A related limitation lies in our study design and analytic plan. We did not anticipate the importance of socializing within the app during our planning process and did not plan for clustering. Because of resource limitations, we were unable to ensure that all participants had equal access to socialization at the same time (ie, some participants had fewer people to talk to in the app for periods of time). We attempted to account for socialization by adding a random effect for cohort into our models, but even that technique cannot truly account for the potential effects of different social opportunities when cohorts do not spend equal time with each other in the app. Future follow-up studies that use the full potential of these apps, which includes online social networking, will need to plan for clustering in their recruitment schedule and analytic plans.

An issue with all studies that use commercially available technology is sustainability. The Jawbone company is no longer manufacturing activity monitors, and it is not clear what the future holds for the Up app. Although other, similar wearables and apps exist, the number of behavior change techniques and the quality of their implementation differ [15]. In particular, social interaction is implemented quite differently in competitor’s products, which could affect future studies’ results.

A possible limitation has to do with the ActivPAL research-grade physical activity measurement devices. Comparisons with other physical activity studies in terms of physical activity time are difficult due to differences in how this outcome is estimated. ActivPAL’s minute estimates are for any physical activity, not only moderate to vigorous intensity activity. Because our focus here is on replacing sedentary time with any kind of activity, we felt this was the appropriate outcome. However, because many other studies use Actigraphs to measure moderate-vigorous intensity activity as their primary outcome, comparisons across studies for active time are difficult. We have provided both steps and active time to allow for more comparisons with other studies.

### Conclusions

An intervention using wearable electronic activity monitors, tablets, and brief phone counseling was found feasible and acceptable in a population of sedentary, overweight middle-aged and older adults. These systems show promise as relatively inexpensive, scalable methods for the delivery of evidence-based behavior change techniques. Future studies are needed to better understand how and why monitor interventions may increase physical activity, for example, by comparing monitors alone with monitors with additional behavior change techniques delivered via counseling.

## Appendix

Multimedia Appendix 1 Behavior change techniques made available by the Jawbone Up system.

Multimedia Appendix 2 CONSORT e-health checklist 1.6.
