# Supplementary material for: Feasibility and Acceptability of a Wearable Technology Physical Activity Intervention With Telephone Counseling for Mid-Aged and Older Adults: A Randomized Controlled Pilot Trial
Source: JMIR Mhealth Uhealth. 2017 Mar 6;5(3):e28. doi: 10.2196/mhealth.6967 (PMC5359416; doi:10.2196/mhealth.6967)
Supplement: Multimedia Appendix 1 [file mhealth_v5i3e28_app1.pdf]

Supplemental File 1.

Behavior change techniques made available by the Jawbone Up system (only those related to physical activity)

Number	Technique name
1.1	Goal-setting
1.2	Problem solving
1.4	Action planning
1.5	Review behavioral goal(s)
1.6	Discrepancy between current behavior and goal
1.9	Commitment
2.2	Feedback on behavior
2.3	Self-monitoring of behavior
3.1	Social support (unspecified)
3.3	Social support (emotional)
4.1	Instruction on how to perform the behavior
4.2	Information about antecedents
5.1	Information about health consequences
5.3	Information about social and environmental consequences
5.4	Monitoring of emotional consequences
5.6	Information about emotional consequences
6.2	Social comparison
7.1	Prompts/cues
8.2	Behavior substitution
9.1	Credible source
10.4	Social reward
12.5	Adding objects to the environment
15.3	Focus on past success

Please note that we cannot determine the extent to which each technique was delivered to any one individual participant. The “SmartCoach” in the Jawbone app tailored content to each user, which means that some participants may not have triggered the app to provide certain types of techniques.

Additionally, please note that these BCTs were systematically determined during a 2014 content analysis (cited in the manuscript). Though we did not find new BCTs in an exploration of the app during the study period, we did not perform another full systematic content analysis. Thus, some BCTs may not have been included.

Behavior change techniques made available in the orientation and counseling calls (this list includes techniques from the CALO-RE taxonomy as well as the hierarchical one so as to include content specific to activity and diet interventions)

Number	Technique name
5	Goal setting (behavior)
7	Action planning (planned specific days/time, length, and potential partners for walks)
8	Identifying barriers/problem resolution
9	Setting graded tasks (encouraged increasing step goals each week)
10	Review of behavioral goals (re-set weekly goal each week)
12	Effort or progress contingent rewards (suggested participants reward themselves)
19	Provide feedback on performance (discussed whether weekly goals were met)
20	Informing when and where to perform the behavior (suggested more, shorter bouts)

23	Training to use prompts (discussion of using the idle alerts)
29	Plan social support
36	Stress management
38	Time management
